# Attributing Illness to Food

**DOI:** 10.3201/eid1107.040634

**Published:** 2005-07

**Authors:** Michael B. Batz, Michael P. Doyle, J. Glenn Morris, John Painter, Ruby Singh, Robert V. Tauxe, Michael R. Taylor, Danilo M.A. Lo Fo Wong, for the Food Attribution Working Group

**Affiliations:** * Resources for the Future, Washington, DC, USA;; † University of Georgia, Griffin, Georgia, USA;; ‡ University of Maryland School of Medicine, Baltimore, Maryland, USA;; § Centers for Disease Control and Prevention, Atlanta, Georgia, USA;; ¶ Food and Drug Administration, Laurel, Maryland, USA;; # Danish Institute for Food and Veterinary Research, Copenhagen, Denmark

**Keywords:** Campylobacter, outbreaks, Risk Assessment, risk factors, Salmonella, organizational models

## Abstract

Identification and prioritization of effective food safety interventions require an understanding of the relationship between food and pathogen from farm to consumption. Critical to this cause is food attribution, the capacity to attribute cases of foodborne disease to the food vehicle or other source responsible for illness. A wide variety of food attribution approaches and data are used around the world, including the analysis of outbreak data, case-control studies, microbial subtyping and source tracking methods, and expert judgment, among others. The Food Safety Research Consortium sponsored the Food Attribution Data Workshop in October 2003 to discuss the virtues and limitations of these approaches and to identify future options for collecting food attribution data in the United States. We summarize workshop discussions and identify challenges that affect progress in this critical component of a risk-based approach to improving food safety.

Foodborne microbiologic hazards may be responsible for as many as 76 million cases of illness in the United States each year (1) and are thus an important food safety challenge. To lower the incidence of foodborne disease, many experts and stakeholders urge the development of a science- and risk-based food safety system, in which decision makers prioritize hazards and interventions using the best available data on the distribution and reduction of risks (2,3). Such a system requires an understanding of the many risk factors between the point of production and the point of consumption and the ability to systematically target intervention efforts along this "farm-to-fork" continuum.

Although the Foodborne Diseases Active Surveillance Network (FoodNet), administered by the Centers for Disease Control and Prevention (CDC), is producing increasingly robust data on the incidence of illness due to specific enteric pathogens, no method exists to categorize these illnesses by mode of transmission, whether drinking water, environmental exposure, or consumption of a specific food. Interventions are almost always food (or process) specific. To design and prioritize effective food safety interventions, we must be able to perform food attribution—that is, identify which foods are vehicles for specific cases of illness. Such data are of particular importance for US government agencies that regulate food and food animals, including the Food Safety Inspection Service (FSIS) of the US Department of Agriculture (USDA), and the Center for Food Safety and Applied Nutrition (CFSAN) and the Center for Veterinary Medicine (CVM) of the Food and Drug Administration (FDA).

Foodborne illnesses can be attributed to foods by using a variety of data sources and analytic approaches; each has its virtues and limitations. In Atlanta on October 31, 2003, the Food Safety Research Consortium (FSRC) sponsored the Food Attribution Data Workshop to explore these approaches in detail. Attendees included representatives from CDC, FSIS, CFSAN, CVM, the Environmental Protection Agency (EPA), consumer advocacy organizations, and member institutions of FSRC, including the University of Maryland at Baltimore, University of Georgia, Iowa State University, University of California at Davis, and Resources for the Future. This article summarizes material discussed at the workshop and identifies challenges that affect progress in this critical component of a risk-based approach to improving food safety.

## Food Categorization

For the purposes of attributing illnesses to foods, food vehicles must be grouped into suitable categories. Although the idea may seem simple, the need for a single food categorization scheme has emerged as a critical issue. At a general level, a list of major food commodities might include 11 categories: poultry, eggs, pork, beef, dairy, fish, mollusks, crustaceans, wild game, row crops (e.g., lettuce and corn), and tree crops (e.g., apples and oranges). Each of these commodities could be divided further, leading to such subcategories as broiler chickens and raw oysters. Additionally, classification could include level of processing (raw, fresh-cut, canned, frozen), origin (domestic, imported), and location of preparation (home, food processor, food service). When illnesses are linked to foods with multiple ingredients, such as soups or casseroles, the choices include whether to omit these cases from analysis, to categorize multiple-ingredient foods by "essential" ingredient, or to attribute illnesses by the proportion of individual ingredients.

A common food categorization scheme is essential if different sources of data are to be combined or compared. Because of lack of agreement in categorization, data from CDC, state health departments, and FDA and USDA and their constituent agencies are often not directly comparable. As a first step in any approach to food attribution, food categories need to be standardized across government agencies, with a scheme acceptable to industry, academia, and consumer groups.

## Current Approaches to Food Attribution

Approaches to food attribution can be grouped into 2 broad categories, loosely designated as epidemiologic and microbiologic. Epidemiologic approaches are based on public health surveillance and include foodborne outbreak data and case-control studies. Microbiologic approaches rely on data on pathogen samples drawn from human, animal, and food sources and include pathogen subtyping, as used in Denmark's Salmonella Accounts (4) and microbial source tracking (MST) methods, as well as risk assessments of specific pathogens in specific foods.

## Danish Experiences

Denmark has an integrated system in which data from public health surveillance and pathogen monitoring of foods and animals are routinely collected, collated, and analyzed by a single coordinating body. Cultures collected from infected persons, animals, and retail food sources are subtyped, allowing for the direct comparison of surveillance and monitoring data and the identification of public health outcomes by food source.

The regular monitoring of food sources is performed on farms, at slaughter, and in retail foods, although the emphasis is on primary production facilities (5). Every flock of egg-laying chickens is regularly tested for salmonellae, as are all flocks of broiler chickens, turkeys, and ducks. Finishing pigs are continually tested, dairy herds are routinely monitored, and poultry, pork, and beef are examined during slaughter processes. Imported meat and poultry products are monitored, as are wild animals, birds, and pets, and retail surveys are performed on raw meat, pork, poultry, shell eggs, fruits, and vegetables.

*Salmonella* isolates obtained from animal and food sources are subtyped (with serotyping, phage typing, and pulsed-field gel electrophoresis [PFGE]) and compared in a quantitative manner with isolates obtained from human infections (6). A prerequisite of the model is predominance of at least 1 "distinctive" *Salmonella* subtype in each main animal reservoir; human infections of distinctive subtypes are assumed to have originated from that reservoir. Human infections caused by *Salmonella* subtypes found in multiple animal reservoirs are attributed proportionally to the occurrence of the distinctive subtypes. Model results have been corroborated by case-control studies, outbreak reports, time-series analysis, and risk assessments (7). In the past 10 years, the Danish model has proven invaluable for identifying pathogen reservoirs in animal populations, tracking trends of human salmonellosis, and guiding interventions (4).

One weakness of the Danish method is that causation cannot be discerned for cases without distinctive *Salmonella* subtypes; thus, the proportional attribution of such cases across animal reservoirs may not necessarily be accurate. Also, vegetables, fruits, fish, pets, water, and other sources of infection are not directly included in the analysis, under the assumption that the original sources of bacterial infection are animal reservoirs. Furthermore, the model is currently focused on salmonellae and may not be applicable to other pathogens that do not meet certain prerequisites. For example, although extensive subtyping has also been performed on *Campylobacter* isolates, the homogeneous distribution of subtypes across reservoirs makes attribution difficult. Since the Danish model is focused on the major food-animal reservoirs, it cannot identify responsible foods at the point of consumption or at other points along the farm-to-fork continuum.

## British Experiences

The United Kingdom uses an integrated systems approach to food safety that includes both epidemiologic and microbiologic methods, with responsibility for foodborne illness consolidated into a single government office. Annual reports on zoonoses, which combine surveillance data with data on food and animal monitoring (8), are produced. In addition, etiologic analyses of foodborne outbreaks, detailing illnesses by pathogen, food source, and additional risk factors, are performed. UK agencies also perform regular pathogen monitoring and subtyping of animals and retail food (8–10).

The United Kingdom has developed a method for estimating the relative risks associated with specific foods, dividing the number of cases due to a specific food (as derived from their outbreak database) by the estimated total servings of that food consumed in a year. The weaknesses of this system include the assumption that all hospitalizations and deaths are routed through general practitioners and reliance on outbreak data, which may not be representative of sporadic disease. However, the UK outbreak dataset is large, and the food vehicles implicated correlate with findings of local epidemiologic studies. Increasingly, data indicate that interventions guided by the system have been successful in reducing cases and risk for foodborne illness.

## US Outbreak Data

Reports of outbreak investigations provide the most comprehensive US data for determining the foods responsible for illnesses. The Foodborne Disease Outbreak Surveillance System contains data on >20,000 US foodborne disease outbreaks reported to CDC since 1973; these reports link specific foods to cases of human illness (11). CDC, the Center for Science in the Public Interest (CSPI), and the FSRC have estimated food attribution using these data (12–16).

Responsibility for investigating foodborne disease outbreaks resides with local and state health departments, which then report these data to CDC. Reported outbreaks represent only a small proportion of those that occur, and the degree of underreporting may vary geographically and temporally. For example, revision of the reporting process and provision of increased resources to CDC and state health departments from the National Food Safety Initiative were associated with a doubling of the number of outbreaks reported annually from 1996 to 1998.

Outbreak data have additional important limitations. Outbreaks that are large, associated with restaurants, have short incubation periods, or cause serious illness are more likely to be investigated and reported. Likewise, illnesses due to pathogens that are difficult to identify or rarely cause outbreaks are underrepresented. For example, the foods most frequently identified as the source of *Campylobacter* outbreaks differ markedly from those identified as sources in community studies of sporadic cases (17).

An approach used by CDC to estimate illness due to a particular food-pathogen combination is to count the number of outbreak-related illnesses due to a particular pathogen and to determine the proportion of these due to each food grouping. These proportions are then applied to estimates of incidence of that pathogen, as reported by CDC (1). This approach is also employed in the Foodborne Illness Risk Ranking Model (FIRRM), an analytic tool developed by FSRC to compare the public health impact of various pathogen-food combinations (14–16). As recent CDC outbreak data were not available when FIRRM was developed, FSRC relied on outbreak data compiled by CSPI. The CSPI database consists primarily (88%) of foodborne outbreak data compiled by CDC and now available on the Internet, but it also includes outbreaks not included in CDC data. CDC may not have received reports on these illnesses from state health departments or may have excluded reports from the database because they did not meet CDC criteria for foodborne outbreaks due to specific pathogens.

Improving food attribution from outbreaks will require improving both the quality and quantity of data. In particular, increased efforts are needed to obtain stool specimens from ill persons early in outbreaks to increase the fraction of outbreaks for which a pathogen is identified to >40% and to trace back foods implicated in outbreaks to their sources. CDC has launched an effort to improve the categorization of food items and ingredients, so outbreaks can be grouped in useful ways for regulatory agencies, industry, and consumers. CDC is also creating new analysis capabilities for the foodborne outbreak surveillance system that will provide data summaries for a variety of purposes, including food attribution.

## FoodNet Sporadic Case-Control Studies

FoodNet is an active surveillance program centered at CDC that tracks foodborne illnesses from 9 pathogens in 10 well-defined target populations (18–20). In FoodNet case-control studies, patients reporting through FoodNet are contacted for followup interviews and to complete questionnaires to estimate the proportion of illnesses associated with specific foods, food preparation, handling practices, and such behavior as pet ownership, farm visits, or international travel. FoodNet has performed case-control studies on a variety of pathogens, including *Salmonella* spp., *Escherichia coli* O157:H7, *Campylobacter*, *Cryptosporidium*, and *Listeria monocytogenes* (among others 17,21–24).

FoodNet case-control studies are of particular value for assessing food attribution of sporadic illness because they are population based. Because the diseases under investigation are rare in all population subgroups, rate ratios in the data closely approximate risk ratios in the population. Along with case exposure percentages, these risk ratio estimates may be used to calculate the "population attributable fraction," the proportion of new cases occurring during a given period in a particular at-risk population that was attributable to the effect of ≥1 exposures.

FoodNet case-control studies have limitations, primarily due to recall bias, long exposure windows, and immunity (20). First, patients and controls are limited in what they remember and can report in an interview, and the interview format itself has limitations. Second, the periods during which exposures are ascertained for FoodNet case-control studies tend to be long (5–7 days), so the likelihood of detecting a difference in exposure between cases and controls is limited by high exposure frequencies among both cases and controls. Further studies are needed to assess the consequence of using shorter exposure windows. Lastly, if a relatively common infection conveys durable immunity, an important segment of the population may be immune and therefore not susceptible to infection, making the demonstration of an association between exposures and risk for infection more difficult.

## Microbial Subtyping and Microbial Source Tracking

CDC's National Molecular Subtyping Network for Foodborne Disease Surveillance (PulseNet) links public health laboratories that use PFGE to routinely fingerprint suspected foodborne bacteria isolates (25). Results of PFGE subtyping of 5 bacteria (*E. coli* O157:H7, nontyphoidal *Salmonella* spp., *Shigella* spp., *L. monocytogenes*, and *Campylobacter* spp.) are stored in the electronic PulseNet database; bacterial strains in the database can be compared quickly and provide an early warning system for emerging outbreaks when related strains appear. PulseNet cannot currently be used for food attribution because it does not include isolates from sporadic cases of human illness or from food or animal sources.

MST refers to a specific application of microbial subtyping in which markers from an isolate are used to trace that isolate to an animal source, similar to what is done for salmonellae in Denmark. If different animal species carry unique, host-specific populations of microorganisms, a subtyped isolate drawn from an infected person could indicate that the isolate originated in 1 species as opposed to another. A large number of MST methods are being researched, most of which were originally developed to trace fecal bacteria in natural waters. Most approaches use genetic or phenotyping fingerprinting methods, although chemical markers, biomarkers, viruses, and bacteriophages are also used as indicators of animal source (26,27). Although MST techniques have potential, no single approach seems ideal for all pathogens and situations. US agencies have begun to research MST specifically for food attribution purposes; CVM, in particular, has investigated methods for *Salmonella* and *Campylobacter* spp (28). Although results to date are promising, they are only initial steps toward using MST methods to attribute illnesses to food animals. Similarly, data collected on the antimicrobial resistance of bacteria by researchers at CVM, CDC, the Agricultural Research Service (an agency of the USDA), and elsewhere may ultimately prove useful for food attribution purposes.

## Risk Assessments

Risk assessments include food contamination data, food storage and consumption patterns, risk behavior, and dose-response functions to predict risks for illness from specific pathogens found in specific foods. If exposure estimates and dose-response functions are sufficiently accurate (a key consideration), risk assessments may produce excellent estimates of the true impact of illness.

Because risk assessments are so resource-intensive, they have been undertaken for only a limited number of pathogen-food combinations. The most comprehensive risk assessments for a single pathogen are those performed for *L. monocytogenes* by CFSAN and FSIS (29,30). This set of 23 individual risk assessments focused on ready-to-eat foods, including deli meats, dairy, produce, and seafood. Risk assessments have also been conducted on *E. coli* O157:H7 in ground beef (31), on *Salmonella enterica* serovar Enteritidis in shell eggs and egg products (32), and on *Vibrio parahaemolyticus* in molluscan shellfish (33).

For risk assessments to be used for food attribution, however, they need to be performed on most food items associated with a particular pathogen. Considering the 3-year duration of the *L. monocytogenes* risk assessments, performing comprehensive risk assessments on a sufficiently large number of pathogen-food combinations for systematic food attribution would be a colossal task.

The other major limitation of using risk assessments for food attribution is that they are inherently predictive. Unlike surveillance data, they do not measure observable public health effects, but rather estimate the impact on the basis of assumptions that are difficult to validate in a dynamic system, in particular dose-response functions, food storage and consumption patterns, and consumer behavior. Furthermore, risk assessments are ill suited for temporal analyses, since they are not routinely updated as new data become available. Risk assessments are most useful for food attribution purposes when compared with other estimates, such as those based on outbreak data or case-control studies.

## US Food Monitoring

Various US food safety agencies test for pathogen prevalence in foods through routine monitoring and case studies. FSIS monitoring, focused on the slaughter process, includes regular testing of raw meat and poultry for salmonellae (34), ground beef for *E. coli* O157:H7 (35), and ready-to-eat deli products for multiple pathogens (http://www.fsis.usda.gov/OPHS/rtetest/). FSIS and the Agricultural Research Service have examined pathogen prevalence in commercial food products, such as *L. monocytogenes* in frankfurters (36). The United States does not have a comprehensive program for monitoring live food animals. Food and animal monitoring data, when not associated with surveillance data, are not applicable for food attribution purposes.

## Expert Elicitation

When scientific or epidemiologic data are lacking, sparse, or highly uncertain, expert judgment may be used to fill gaps or combine conflicting estimates into a meaningful solution. Expert judgments derived through formal methods are increasingly used and recommended for assessing risk and the economic impact of regulations (37–39).

FSRC researchers administered an expert elicitation of experienced food safety researchers, public health scientists, and food safety authorities for use in the Foodborne Illness Risk Ranking Model (15). Produced with a standardized, vetted method, the survey asked respondents to estimate the percentage of 11 pathogens caused by each listed food category and included measures of respondent uncertainty and possible biases. Although data need to be analyzed further, initial results are promising and corroborate food attribution percentages derived from other means.

Expert elicitations are limited because they are based on perception, not on observable data. Results may be circular if experts rely on the same sources, or deceptive if experts are similarly misinformed or biased. Expert judgments are thus not an ideal source of food attribution data but may have utility if data are sparse or inconsistent and uncertainty is substantial.

## Conclusions

A recent National Academies of Science report, Scientific Criteria to Ensure Safe Food, argues for "the development of a comprehensive national plan to harmonize the foodborne disease surveillance that is conducted by public health agencies with the monitoring of pathogens across the food production, processing, and distribution continuum that is conducted by food safety regulatory agencies" (2). The motivation driving this suggestion is the same as that which motivated FSRC to convene the Food Attribution Data Workshop; to make informed science- and risk-based decisions about food safety interventions, we need to be able to associate foodborne illnesses to specific food vehicles. The goal of the workshop was to review the approaches currently used for food attribution, in the United States and abroad, and to identify future options for the collection of food attribution data in the United States.

Although all workshop attendees or institutions did not reach consensus about the ideal data for food attribution, there was nearly universal agreement that none of the current data sources are sufficient on their own because of methodologic limitations or gaps in available data (see Table). Furthermore, in the United States, data are spread over a wide range of agencies and researchers, resulting in myriad studies covering different aspects of the food attribution problem. These issues make it difficult to accurately and dependably attribute illnesses to the foods responsible as pathogen vehicles—and, in turn, to target appropriate intervention strategies.

**Table:** Current approaches to food attribution

Approach	Primary advantages	Primary limitations	Refs
Denmark Salmonella Accounts	Microbial subtyping provides direct link between public health endpoint and animal	Difficult to expand to other pathogens; requires distinctive subtypes across reservoirs	5,6
	High reporting of illnesses (social health care)	Focus on animals ignores nonanimal sources	
	National, temporal coverage for both illnesses and animal/product monitoring	Focus on reservoirs, not food products at point of consumption	
UK outbreak data	Large dataset: national, temporal coverage	May not correlate with sporadic case data	8
	Results correlate with local epidemiologic findings	Not all pathogens well represented	
		Dependence on general practitioners	
US outbreak data	National and temporal coverage	May not correlate with sporadic case data	11,12
	Large common dataset	Geographic and temporal inconsistencies (local reporting) and biases towards certain foods	
	Straightforward, uses existing data	Not all pathogens well represented	
	Outbreaks and outbreak cases can be aggregated into food categories		
Case-control studies	Population-based studies	Survey format has recall bias and other limits	17,20–24
	Captures risk factors not included in most surveillance data (travel, food preparation questions)	Long exposure windows (problems with common exposures)	
	Can implicate risks missed by laboratory testing	Durable immunity in population can impede associating exposures with illnesses	
		No laboratory verification	
Microbial subtyping	Subtyping of illnesses and foods can provide direct link between public health endpoint and source of infection	For animal sourcing, subtypes must be distinctive across species (see Danish Salmonella Accounts)	25–28,5,6
	Can be used to identify specific foods (outbreak investigations) or animal reservoirs (source tracking by species)	Utility may be limited to certain pathogens	
	Many different techniques, growing fast	Resource intensive; requires human surveillance, extensive monitoring of food and animals, plus laboratory testing, data storage, analysis	
Risk assessments	Can estimate cases not captured by surveillance methods (not limited by underreporting or biases in epidemiologic methods)	Predictive; cannot be verified	29–33
	Uses consumption and contamination data ignored by surveillance-based approaches	Large uncertainties in dose-response models and exposure estimates	
		Resource- and time-intensive (each pathogen-food combination requires its own exhaustive study)	
Food monitoring data	Captures upstream contamination (avoids environmental and cross-contamination after purchase)	Not usable for food attribution unless made compatible (through subtyping or other means) with public health data	34–36
Expert elicitation/judgment	Useful when data are sparse or conflicting	Respondents can be similarly biased	15,37–39
	Formal methods increase utility	Requires some level of consensus for reasonable error bounds	
		Based on perception, not data	

Several characteristics should be considered in evaluating and comparing current and future food attribution methods; their relative importance depends on the purpose for which the attribution data are sought. These include scientific accuracy and uncertainty, quality and breadth of data, computational consistency, practical feasibility, cost of implementation, flexibility and scalability, utility for targeting interventions, and congruency with other relevant data sources. Among the critical unresolved issues is how to balance such factors as scientific accuracy and practical feasibility to produce attribution data that will be both useful and affordable.

With so many institutions responsible for various aspects of the food safety system, collaboration is paramount, as is the explicit delineation of responsibilities and powers. Access to these data is a critical issue. Building a system in which data and conclusions are shared in a timely manner among agencies and with industry and academia, and privacy issues with persons and industry participants have to be addressed. Creation of an open searchable database of outbreaks would greatly expand the opportunities for research and collaboration.

As described here, a variety of approaches have been used to better define the source of foods responsible for human infections. However, none of these approaches is likely to be sufficient on its own. The implicit conclusion, therefore, is that the scientific and accurate attribution of foodborne illnesses to specific foods means developing a comprehensive program that combines many of the discussed methods and data. Such a system can be achieved with increased resources and cooperation among food safety institutions.

The Food Attribution Data Workshop was sponsored by the Food Safety Research Consortium, a multidisciplinary collaboration to improve public health; members include the University of Maryland, Baltimore; University of Georgia; Iowa State University; University of Massachusetts; University of California, Davis; Michigan State University; and Resources for the Future. The workshop grew out of an FSRC project funded by the Robert Wood Johnson Foundation and received generous support from the Joint Institute for Food Safety and Applied Nutrition, the Center for Food Safety and Applied Nutrition and Center for Veterinary Medicine within the FDA, and the Office of Risk Assessment and Cost-Benefit Analysis and the Economic Research Service within USDA.
